# Hydroxytyrosol in the Prevention of the Metabolic Syndrome and Related Disorders

**DOI:** 10.3390/nu9030306

**Published:** 2017-03-20

**Authors:** Julien Peyrol, Catherine Riva, Marie Josèphe Amiot

**Affiliations:** 1 Laboratory of Cardiovascular Pharm-Ecology EA4278, Department of Sport Sciences, Faculty of Sciences, Avignon University, F-84000 Avignon, France; julien.peyrol@univ-avignon.fr; 2 Unité Mixte de Recherche (UMR), Nutrition, Obesity and Risk of Thrombosis, Aix-Marseille University, F-13005 Marseille, France; 3 Unité Mixte de Recherche (UMR), Markets, Organisations, Institutions, Stakeholder Strategies, F-34060 Montpellier, France; 4 Centre de Coopération Internationale en Recherche Agronomique pour le Développement, F-34060 Montpellier, France; 5 Centre International de Hautes Études Agronomiques Méditerranéennes, F-34060 Montpellier, France; 6 Montpellier SupAgro, F-34060 Montpellier, France; 7 Institut National de la Recherche Agronomique; Division of Nutrtition, Chemical Food Safety and Consumer Behaviour, F-75015 Paris, France; 8 Institut National de la Santé et de la Recherche Médicale, F-75015 Paris, France

**Keywords:** olive oil, oleuropein, hydroxytyrosol, tyrosol, body weight, dyslipidemia, hyperglycemia, hypertension, oxidative stress, inflammation

## Abstract

Virgin olive oil (VOO) constitutes the main source of fat in the Mediterranean diet. VOO is rich in oleic acid, displaying health-promoting properties, but also contains minor bioactive components, especially phenolic compounds. Hydroxytyrosol (HT), the main polyphenol of olive oil, has been reported to be the most bioactive component. This review aims to compile the results of clinical, animal and cell culture studies evaluating the effects of HT on the features of Metabolic Syndrome (MetS) (body weight/adiposity, dyslipidemia, hypertension, and hyperglycemia/insulin resistance) and associated complications (oxidative stress and inflammation). HT was able to improve the lipid profile, glycaemia, and insulin sensitivity, and counteract oxidative and inflammatory processes. Experimental studies identified multiple molecular targets for HT conferring its beneficial effect on health in spite of its low bioavailability. However, rodent experiments and clinical trials with pure HT at biologically relevant concentrations are still lacking. Moreover, the roles of intestine and its gut microbiota have not been elucidated.

## 1. Introduction

Metabolic syndrome (MetS), a cluster of several interrelated cardiovascular risk factors (hyperglycemia, hypertension, dyslipidemia, insulin resistance and central adiposity) [1] lead to an increased prevalence of cardiovascular diseases (CVD) and type 2 diabetes mellitus (T2DM). A report published in 2015 claimed that the mortality for T2DM is around five millions persons per year, and it is expected that 23.6 million will die of CVD in the world by 2030 [2]. Olive oil, a natural juice from olive, is the primary source of fat in the Mediterranean diet, which is associated with a lower incidence of CVD mortality [3] and contains minor components, especially phenolic compounds, which are recognized as health beneficial components [4]. Hydroxytyrosol (HT), a non-flavonoid polyphenolic compound derived from oleuropein (OLE) and, notably present in olive and olive oil, could be involved in lower incidence of CVD and T2DM in the Mediterranean region, despite a high intake of fat as olive oil. HT could be an efficient bioactive phenolic candidate, owing to its protective action of health towards inflammation and oxidative stress. Despite its well-described actions, the low plasmatic concentrations of HT after 25 mL extra-virgin olive oil (EVOO) consumption, ranging from 50 to 160 nM [5,6] questioned the assessment of its bioactivity. In addition, among the exponential increase of published studies on HT, few are related to its effects on MetS key components and the associated complications. Herein, this review discusses the effects of HT on MetS key components and the molecular mechanisms exerting its health protective effects.

## 2. Mediterranean Diet and Olive Oil as Primary and Secondary Preventive Nutritional Strategies

The Mediterranean diet pattern is characterized by a high consumptions of fruits, vegetables, beans, nuts, unrefined grains, and fish; a lower intake of meats and full-fat dairy products; and a daily consumption of olive oil as the mostly used fat in culinary practices. Fatty acid consumption, characterized by a higher rate of monounsaturated (MUFAs) and polyunsaturated (PUFAs) fatty acids consumption than saturated fatty acid (SFAs), is central in the consumption of VOO in the Mediterranean population countries. Indeed, Greece, Italy and Spain are characterized by a higher consumption of MUFAs compared to SFAs, whereas, in the US, the ratio MUFA/SFA is ~1 [7,8].

Lifestyle interventions using a Mediterranean-type diet reported inverse associations between a good adherence to this pattern and the risk of CVD [9] or T2DM [10] and showed a reduction in the incidence of key components of MetS including obesity [11,12,13], hypertension [14,15,16], glucose tolerance [17], dyslipidemia [18] and insulin resistance [19]. Olive oil, the main MUFAs in the Mediterranean diet, has been widely identified as the initiator of these health benefits with increasing consumption of virgin olive oil (VOO) enhancing lipid profile, reducing blood pressure and endothelial dysfunction, improving inflammatory and prothrombotic environment through reduced Low Density Lipoprotein (LDL) oxidizability [20]. Recently, the Prevencion con Dieta mediterránea study (PREDIMED) [21] showed that patients with a Mediterranean diet supplemented with extra-virgin olive oil (EVOO) or nuts had a lower incidence of T2DM and a reduced associated-mortality. EVOO provided in the Mediterranean diet was beneficial for blood pressure, glycemia, dyslipidemia, oxidative stress and inflammation [22,23]. Interestingly, the EUROLIVE (Effect of Olive Oils on Oxidative Damage in European Populations) study highlighted inverse relationships between the total cholesterol/High Density Lipoprotein (HDL)-cholesterol ratio or oxidative stress markers and the phenolic content of the olive oil [24]. The cardio-protective actions of olive oil components have been reported [25,26,27] and the non-saponifiable minor bioactive compounds [28,29,30] such as phenolic compounds including HT and its precursor OLE, rather than oleic acid, were reported responsible for the protective properties [31].

## 3. Olive Oil Composition and Polyphenolic Fraction

The olive oil composition can be divided into two fractions, saponifiable and unsaponifiable fractions. The saponifiable fraction of olive oil corresponds to the total amount of SFAs, MUFAs and PUFAs. Olive oil is characterized by a high content of MUFAs, whereas the concentrations of SFAs and PUFAs range from 8% to 26% and from 3% to 22%, respectively. Oleic acid is present up to 83% in virgin olive oil [32].

The unsaponifiable fraction contains more than 200 compounds; among them, phenolic compounds account for 3% of the total oil composition [33]. This fraction contributes to the specific characteristics of olive oil, such as aroma, taste, color and oxidative stability [34]. The most abundant fraction of the unsaponifiable fraction is hydrocarbons (squalene, *β*-carotene and lutein). Other compounds are phytosterols, triterpenic compounds in the form of dialcohols or acids and fat-soluble phenols including tocopherol and tocotrienols. The most polar fraction consists in phenolic compounds, which can be classified into several groups: phenolic acids (caffeic, ferulic, gallic, gentisic, *o*-coumaric, *p*-coumaric, *p*-hydroxybenzoic and protocatehuic acids), phenolic alcohols 3,4-dihydroxyphenylethanol named HT and tyrosol (Tyr), phenolic secoiridoids, hydroxyl-isocromans (formed by the reaction between HT and benzaldehyde or vanillin), flavonoids and lignans [35]. In olive fruit, phenolic compounds are present as glycosylated forms. Oleuropein aglycone is a phenolic secoiridoid liberated from the glucoside form, OLE, upon the action of a *β*-glucosidase during olive ripening. In olive oil, oleuropein aglycone is degraded into elenolic acid, the secoiridoid moiety, and HT, the phenolic moiety.

## 4. Virgin Olive Oil Phenols Concentration

Not less than 30 polyphenols have been identified in olive oil and considerable variations have been noted in the concentrations of these phenolic compounds. Phenolic concentration in EVOO ranges from 50 to 800 mg/kg [36] with a mean of 230 mg/kg, whereas in refined olive oil it is much lower [37].

HT and their corresponding secoiridoid derivatives constitute around 90% of the total phenolic content of VOO [38]. Olive oil phenol concentration depends of olive variety [39], agricultural environment and practices [39], the maturity stages of the fruit [39], storage conditions and processing [40,41].

## 5. Bioavailability of Hydroxytyrosol and Metabolism

A consensus indicated that HT does not exert any cytotoxic effect on cells [42], or animals [43,44]. In 2000, Visioli et al. [45] showed that olive oil phenols, especially HT and Tyr, are dose-dependently absorbed in humans after ingestion and excreted in urine. However, the levels of free HT and Tyr in urine were lower compared with their glucuronide metabolites. In addition to glucuronides, other metabolites of HT were found in plasma and urine such as the methylated or sulfated forms [46]. It has been reported that there is a significant absorption (40%–95%) of HT [45,46,47,48] indicating that human intestine absorbs a major part of ingested VOO phenolic compounds. Moreover, HT is more assimilated when given as an olive oil compared to an aqueous solution [49], and its absorption was greater when ingested in its natural form present in extra-virgin olive oil rather than added in refined olive oil or incorporated into a yoghurt, as shown by its urinary recoveries being 44%, 23% and 5.8%, respectively [50]. Such results suggested that the olive oil matrix could act as a protective factor preventing the degradation of phenolic compounds in the gastrointestinal tract. The range levels of circulating metabolites of OLE was 10–60 µg post-ingestion of a 50 mL high-phenol-containing VOO [51,52].

Consumption of VOO in the Mediterranean countries is expected to be around 30–50 g/day [53] leading to an intake of 200 µg of phenolic compounds. Taking into account that the absorption rate of phenols is in the range of 40%–95%, it results an amount of 4–9 mg/day of olive oil phenols. Note that, in the gastrointestinal tract, HT and Tyr result from the degradation of aglycones of OLE and its monophenolic form ligstrosides. Its degradation is incomplete and OLE can be readily absorbed across the intestine [54] by possible implication of glucose transporter [55]. Besides, glucuronidation of HT was previously reported in intestinal Caco-2 and in HepG2 cells [56,57,58].

Note that D’Angelo et al. [43] have found that intravenous administration of HT led to a fast and extensive uptake of this molecule within 5 min after injection in several tissues such as skeletal muscle, heart, liver, lungs and kidney.

## 6. Hydroxytyrosol, Body Weight and Development of Adipose Tissue

Body weight is the main outcome to define obesity and body mass index increase is positively correlated with MetS. Only one clinical study assessed the effect of HT supplementation on body weight showing that 12-week supplementation of HT (9.67 mg/day) associated with OLE (51.1 mg/day) did not exert any effect on body weight in overweight men [59]. The absence of effect was confirmed in numerous rodent experiments [60,61,62,63,64], except for one study that showed a beneficial effect of HT (50 mg/kg/day × 17 weeks) supplementation against diet-induced obesity [65].

Whereas no in vivo studies experienced the impact of VOO phenolic compounds on adipose tissue development, in vitro, VOO phenolic compounds have been shown to influence adipocyte hyperplasia and hypertrophy through the expression of genes related to obesity. It was reported that HT (25 and 150 µM) reduced hyperplasia and hypertrophy by reducing triglycerides content by downregulating adipogenesis-related genes, peroxisome proliferator-activated receptors α and γ, peroxisome proliferator-activated receptor γ coactivator 1-α (PGC-1α), lipoprotein lipase, hormone sensitive lipase, acetyl CoA carboxylase-1, carnitine palmitoyltransferase-1, CCAAT/enhancer binding protein α, and sterol regulatory element-binding transcription factor-1 transcription factors and downstream genes (glucose transporter-4, CD36 and fatty acid synthase) [66,67]. Taken together, these data suggested that HT might reduce the size of adipocytes and be beneficial for reducing the risk of obesity.

The processes of adipose hypertrophy and hyperplasia are associated with mitochondrial stress and dysfunction observed by a reduction of adenosine triphosphate (ATP) formation and a reduction of mitochondrial complex expression subunits. In a murine model of high fat diet (HFD)-induced obesity, it has been shown that HT (50 mg/kg/day × 17 weeks) could normalize mitochondrial complex subunit expression and mitochondrial fission marker dynamin-related protein-1 [65]. The high amount of subunit expression could be attributed to an enhancement of mitochondria quantity. Indeed, Hao et al. [68] have found that HT allows to enhance Mitochondrial transcription factor A (1 µM), Nuclear respiratory factors 1 and *2* (Nrf1 and Nrf2) (1 µM) mRNA, key activators of mitochondrial transcription and genome replication, thus increasing protein levels of complexes 1, 2, and 3 in adipocytes (0.1, 1 and 10 µM). The authors also found an increase of peroxisome proliferator-activated receptors α and γ (1 and 10 µM) and carnitine palmitoyltransferase I (1 and 10 µM) expressions, which are implicated in mitochondria biogenesis, suggesting a possible better oxidative status in adipocytes.

Furthermore, it seems that HT (1 µM) acts as a starving agent, since an increase in adenosine monophosphate kinase (AMPK), acetyl CoA carboxylase, hormone-sensitive lipase and lipase phosphorylation were reported in adipocytes [68].

## 7. Hydroxytyrosol and Lipid Metabolism

The prospective EUROLIVE study demonstrated that olive oils with different levels of polyphenols led to a reduction in LDL-c and TG [24] in a dose-dependent manner. The absence of body weight gain suggested that HT could possess a lipolytic function, especially in adipose tissue. Whereas clinical trials were not undertaken, some experimental studies, performed in rodent and murine models (0.03% × 8 weeks and 50 mg/kg/day × 17 weeks) [63,65] or adipocytes (150 µM and 1 µM) [66,68] reported that HT attenuates TGs accumulation in adipocytes, blood, liver and skeletal muscles (50 mg/kg/day × 17 weeks and 25 µM) [65,69]; glycerol release (75 µM) [69]; and lowers serum cholesterol in HFD-rats (10 mg/kg/day × 5 weeks) [70], and LDL and HDL-c levels (50 mg/kg/day × 17 weeks) [65] and plasma cholesterol in control rats (0.03% × 8 weeks) [63]. Moreover, HT treatment inhibited epididymal and perirenal fat formation and limited liver weight gain (50 mg/kg/day × 17 weeks) [65]. On the other hand, it has been demonstrated in a *db/db* model of mice, that HT (10 mg/kg/day × 8 weeks) increased the activity of mitochondrial complex, and lipolysis fatty acid oxidation-related genes [65]. In contrast, Acin et al. [71] reported that HT (10 mg/kg/day × 10 weeks) had deleterious effects with increasing plasma cholesterol, very low density lipoprotein-cholesterol, and LDL-c and reducing ApoA-1 resulting in an increased atheroma plaque formation.

In vitro, HT was reported to increase oxygen consumption, suggesting a higher oxidative rate to produce ATP [65], proteins implicated in mitochondria biogenesis, mitochondria mass and size [68]. AMPK was decreased during chronic stress situation, thus reducing glycolysis and fatty acid oxidation. Moreover, Cao et al. [65] have reported, in obese mice, that HT supplementation (50 mg/kg/day × 17 weeks) leads to a reduction in SREBP-1c level, a well-known regulator of fatty acid and cholesterol synthesis in liver.

## 8. Hydroxytyrosol, Glucose Homeostasis and Insulin-Resistance

The strength of olive oil to reduce the incidence of all the glucose-associated disorders is no longer to be demonstrated. Moreover, the enhancement of glucose tolerance was shown to be dependent of the concentration of polyphenols and olive oil [72]. Clinical trials regarding the impact of HT on carbohydrate metabolism are still lacking but experiments in rodent models of MetS are available and suggested that HT is able to reduce plasmatic glucose concentration (50 mg/kg/day × 17 weeks, 20 mg/kg × 2 months and 0.04% × 8 weeks) [65,73,74] and insulin secretion (50 µg/mL) [73] leading to a decrease of insulin-resistance [65,74]. Moreover, Pirozzi et al. [70] found that HT (10 mg/kg/day × 5 weeks) enhances glucose tolerance and increases insulin sensitivity leading to a decrease of homeostatic model assessment-insulin resistance. Interestingly, in a *db/db* model of mice, Cao et al. [65] have reported that HT given at 10 mg/kg/day for 8 weeks decreases fasting glucose level.

## 9. Hydroxytyrosol and Hypertension

Clinical trials have demonstrated that olive oil is more efficient than any other oil at reducing blood pressure [75,76,77,78]. It has been hypothesized that the effect of olive oil on blood pressure was not only mediated through its MUFAs content but also through its polyphenol content. Indeed, some studies mentioned that the polyphenols of olive oil were responsible of the anti-hypertensive effect of olive oils, as demonstrated in hypercholesterolemic [79] or pre-hypertensive subjects [80] after consuming polyphenols enriched olive oil. Ruiz-Gutierrez et al. [81] reported a reduction of both systolic (SBP) and diastolic (DBP) blood pressures after an olive oil-rich diet but not after a high-oleic-acid sunflower diet. In this sense, clinical trials proved that consumption of OLE was able to reduce SBP and DBP after consumption of OLE in both pre-hypertensive subjects (136 mg/day + 6 mg/day HT × 6 weeks) [82] and hypertensive rats (30 mg/day × 5 weeks) [83]. Given the fact that OLE is degraded into HT, the question arose if the blood pressure lowering effect was due to OLE or HT. Lopez-Villodres et al. [84] found that HT supplementation (10 mg/kg/day × 2 months) increased in diabetic rats the levels of nitrites and nitrates, potent donors of NO acting as vasorelaxing agent. In addition, Storniolo et al. [85] demonstrated that HT (10 µM) counteracted hyperglycemia-induced endothelin-1 expression, a well-known hypertensive agent, in a more extend than oleic acid.

## 10. Associated Complications: Oxidative Stress, Inflammation and Cardiovascular Dysfunction

### 10.1. Antioxidative Properties

Oxidative stress is a central physiologic process playing an important role in the maintenance of intracellular homeostasis. However, despite intracellular protective mechanisms, including superoxide dismustase (SOD), Catalase (Cat) and reduced glutathione, excess reactive oxygen species (ROS) is detrimental to cellular physiology. Obesity and T2DM are characterized by an excessive amount of ROS overwhelming intracellular defenses and leading to reinforce MetS associated complications. Polyphenols have been used as nutraceutical antioxidant for several years since an increased amount of fruits and vegetables were linked to the reduction of oxidative pathologies.

#### 10.1.1. Hydroxytyrosol and LDL Oxidizability

Oxidation of the lipid part of LDL leads to a change in the lipoprotein conformation by which LDL is better able to enter into monocytes/macrophage of the arterial wall and develop the atherosclerotic process. Human studies suggested that olive oil protects LDL against oxidation as indicated by decreased LDL oxidizability [86,87] and this strong effect prevails on linoleate-rich particles [88]. It has been well demonstrated that phenolic compounds, especially HT, are protective against LDL oxidation. Based on this protective effect, the European Food Safety Authority claimed that 5 mg of HT (as free and derived forms) should be consumed daily. To prove that HT is efficient, its supplementation (45–50 mg/day × 3 weeks) in sunflower oil was shown to reduce oxLDL [89], suggesting that HT could prevent CVD. These results in clinical trials were corroborated by animal experiments [74,90]. Increase of lag-time [91,92] is the main outcome of reduction in LDL oxidizability and this change could be attributed to an increase in oleic acid [88] or HT [38,60,93] rate in LDL. Such mechanism occurs rapidly and increases with phenolic compounds in olive oil [94].

Mateos et al. [95] reported that consumption of polyphenol-rich VOO leads to a reduction of the expression of pro-atherogenic genes such as CD40 antigen ligand and oxLDL receptor-1 when compared with the refined olive oil, which was depleted in polyphenols [87]. Another mechanism that can be implicated in the protection of LDL by olive oil could be the enhancement of arylesterase plasma activity, an enzyme presents on HDL surface, suggested to contribute to the antioxidant protection conferred by HDL on LDL oxidation [96,97]. However, the scavenging properties of HT cannot be excluded in the protection of LDL oxidizability (10 µM) [98,99]. In fact, Briante et al. [100] reported that HT protects, in vitro, LDL from oxidation at a concentration >18 µg/mg of LDL.

#### 10.1.2. Hydroxytyrosol and Mitochondria

There is evidence that mitochondrial dysfunction in MetS is associated with T2DM [101,102]. Genetic factors, oxidative stress, mitochondrial biogenesis and aging may affect mitochondrial function, leading to insulin resistance. Fewer and smaller-sized mitochondria have been found in skeletal muscles of insulin-resistance, obese, or T2DM subjects and are linked with a lower mitochondrial oxidative capacity [103]. The decreased mitochondrial oxidative capacity is associated with the reduction in expression of mitochondrial genome [104]. To counteract such effect and enhance oxidative metabolism, HT was supposed to be a good candidate. Although no clinical trial investigated the effect of HT supplementation, studies in obese- and diabetic-rendered rats and in doxorubicin-induced cardiotoxicity rats revealed that HT (0.5, 10 and 50 mg/kg/day) is able to increase mitochondrial function through an enhancement of mitochondrial complex subunit expression [65,105] and activity [105,106]. Enhanced mitochondrial activity was associated with an increase of uncoupling protein-2 protein expression (100 µM) [107]. All the animal experiments supported the impact of HT in the protection of mitochondria from oxidative damages, which operates a shift towards a more efficient oxidative metabolism. Furthermore, it has been demonstrated that HT increases the mitochondrial deoxyribonucleic acid content (1 µM and 10 and 50 mg/kg/day) [68,108], the mitochondria function and membrane potential (0.1 and 10 µg/mL) [109] and density (1 µM) [68]. Mitochondrial biogenesis and respiration were stimulated by PGC-1α by strongly inducing its gene expression. Rodent and culture cell experiments reported also that HT was able to increase PGC-1α and Nrf2 expression (0.1, 1 and 10 µM and 100 µM) [68,107] and AMPK, an upstream regulator of PGC-1α [68,107]. Note that an increase in maximal oxygen consumption was found (1 and 10 µM) [68].

#### 10.1.3. Hydroxytyrosol and Antioxidant Protein Expression

There is a recognized link between oxidative stress and key components of MetS. Besides LDL oxidation, other oxidative markers also showed improvements. HT was reported to prevent the increase of protein carbonyl levels and lipid peroxidation markers, and to normalize liver glutathione level, liver glutathione *S*-transferase, and total SOD activity in obese mice (10 and 50 mg/kg/day) [65]. In cell culture, it was also demonstrated that HT increases Cu/SOD expression (100 µM) [107]; normalizes glutathione concentrations; increases glutathione peroxidase, glutathione reductase and glutathione-*S*-transferase protein expression (0.5 to 10 µM) [110]; increases CAT activity (50 µM) [111]; and reduces the reduced glutathione/oxidized glutathione (known as GSH:GSSG ratio) (1 and 5 µM) in presence of hydrogen peroxide, suggesting a reduction in the oxidative status [63]. The antioxidative capacities are not limited to the expression of type 2 detoxifying proteins as SOD, Cat, and glutathione peroxidase. Indeed, it exists adaptive systems as those implying heme oxygenase-1, the expression of which is regulated by Nrf2. The positive impact of HT on Nrf2 nuclear translocation was shown and associated to phosphoinositide/protein kinase B and extracellular signal-regulated kinase pathway (0.5 to 10 µM) [110] and also AMPK/forkhead box 3a (50 µM) [111].

Moreover, it has been found that HT (20 µg/day) for four weeks was able to reduce plasma hydroperoxide concentrations, normalize plasma malondialdehyde and conjugated dienes, and increase plasma antioxidant capacity in a rat model of HFD-induced obesity [64].

#### 10.1.4. Hydroxytyrosol and Superoxide (O_2_^•−^) Scavenging Properties

In an acute model of oxidative stress driven in rat aortas, Rietjens et al. [98] showed that HT acts as a scavenging agent. Since, numerous studies were published and confirmed that HT protect against ROS production in human vascular endothelial cells, erythrocytes and renal epithelial tubular cells (5 to 80 µM) [111,112,113], displays scavenger activity for peroxynitrous acid (5 µM to 1 mM) [114,115] and has a protective role on deoxyribonucleic acid damages associated to peroxinitrous acid (0.05 to 1 mM) [114]. Moreover, it can inhibit superoxide anion burst from macrophages but not from neutrophils where it can only scavenge hydrogen peroxide (1 to 50 µM) [116]. It also protects erythrocytes from hemolysis (50 to 200 µM) [117], endothelial cells from monocytes adhesion (0.5 to 2.5 µM) [118] and hepatocytes (10 to 40 µM) [119], and protects from lipid peroxidation in rat livers (5 to 60 µM) [120] as well as from lipid oxidation (10 µM) [91].

### 10.2. Hydroxytyrosol and Inflammation

It is well known that the pathophysiology of MetS causes chronic inflammation. HT has been reported to possess significant anti-inflammatory capacity. In fact, in clinical trial, HT (25 mg/day × one week) led to a reduction of plasma CRP and isoprostane levels, but did not exert any effect on other inflammatory markers as interleukin-6, monocytes chemoattractant protein-1 and tumor necrosis factor-α (TNF-α) [121]. In rodent experiments, it was demonstrated that HT reduces TNF-α, IL-6 and cyclooxygenase-2 expression in liver (50 mg/kg/day × 17 weeks) [65], increases the anti-inflammatory IL-10 expression (12.5 µg/mL and 10 mg/kg/day × 10 days) [122,123], reduces inducible nitric oxide synthase (iNOS) expression (12.5 µg/mL and 5 mg/kg/day × 30 days) [122,124,125] and cyclooxygenase-2 expression [125]. Considering that leptin, a well-known protein acting on satiety and having inflammatory property, HT was shown to lower leptin level in mice thus suggesting that could act as a starving and anti-inflammatory agent (0.03% × 8 weeks and 50 mg/kg/day × 17 weeks) [63,65] and attenuate TNF-α and IL-1β expression in animal model [125,126]. In vitro, HT has been reported to increase adiponectin expression and secretion in the presence of TNF-α (1 to 20 µM) [127], reduce iNOS, cyclooxygenase-2 and TNF-α expressions (25 to 100 µM) [128], reduce nuclear factor-κB (NF-κB) binding activity (50 and 100 µM) [129] and, interestingly, increase iκBα expression, an inhibitor of NF-κB binding activity [129]. Moreover, a reduction of metalloproteinase-9 activity and secretion (1 and 10 µM) [130] and a reduction of prostaglandin E_2_ secretion and expression were found (1 and 10 µM) [130].

### 10.3. Hydroxytyrosol and Atherosclerosis

Phenolic compounds in VOO were shown to improve endothelial dysfunction and reduce oxidative stress plasma parameters, both playing a key role in the development of atherosclerosis [131,132]. Moreover, VOO phenolic compounds could counteract inflammation, which is an important trigger in the development of atherosclerosis though the expression of adhesion molecules. In a clinical trial enrolling healthy volunteers, it has been demonstrated that sunflower oil supplemented with HT decreased vascular cell adhesion protein (VCAM-1) plasmatic concentration (45–50 mg/kg/day × 3 weeks) [89] and monocyte chemoattractant protein-1 and interleukin-8 receptor expression (366 mg/kg/day × 3 weeks) [87]. Such results were confirmed in rodent models, where HT reduced platelet aggregation, VCAM-1 and IL-1β expressions (0.5 to 10 mg/kg/day × 2 months) [84] and TNF-α expression (0.04%) [74]. Interestingly, Gonzalez et al. [60] reported that HT (4 mg/kg/day × 2 months) reduced the size of atherosclerotic lesions in rabbits fed with a high fat and high cholesterol diet.

Several in vitro studies confirmed the anti-inflammatory capacity of HT by reducing VCAM-1, intercellular adhesion protein expressions (0.5 to 75 µM) [22,118,133]. Molecular mechanisms leading to this reduction probably involved the reduction of NF-κB activation (0.5 to 75 µM) [22,23]. Despite the great interest surrounding HT as a nutraceutical, Acin et al. [71] reported that HT supplementation (10 mg/kg/day) for 10 weeks led to an increase in atherosclerotic plaque, in monocyte activation and a reduction in ApoA-I from HDL in ApoE-deficient mice. However, these mice did not develop obesity, low-grade inflammation and oxidative stress, thus explaining this deleterious effect because HT could act as an oxidant.

### 10.4. Hydroxytyrosol and Vascular Dysfunction

Nitric oxide (NO^•^) plays a pivotal role in endothelial function and its decreased bioavailability is correlated with altered vascular tone. Lopez-Villodres et al. [84] found that in streptozotocin-induced model of diabetes, HT supplementation (10 mg/kg/day × 2 months) increased the level of nitrates and nitrites. HT was reported to be ineffective on eNOS expression and activity (i.e., phosphorylation on its Ser1177) in absence of oxidative stress in HUVECs (0.1 to 100 µM) [134]. In an endothelial cell culture model of hyperglycemia, Storniolo et al. [85] showed that HT (10 µM) increases NO^•^ production, which is correlated to an increase of endothelial nitric oxide synthase phosphorylation (P-eNOS)/endothelial nitric oxide synthase (eNOS) ratio. When stimulated by acetylcholine, an activator of eNOS/NO^•^ signaling pathway, HT increased NO^•^ production, more than oleic acid; this increase was linked to higher intracellular calcium concentration. Rietjens et al. [98] evidenced that HT (10 µM) enhances endothelium-dependent relaxation in addition to increase P-eNOS/eNOS ratio. This enhancement was associated with a cGMP increase, a downstream molecule of eNOS acting on smooth muscle cells relaxation. In a vascular endothelial cell culture model, Zrelli et al. [135] found that HT (50 µM) increases P-eNOS resulting in increasing NO^•^ production, associated to the decreased of NF-κB and iκBα phosphorylation in the presence of TNF-α. These results suggest that eNOS could decrease inflammatory response, and thereafter, decrease thrombus formation. Furthermore, it was demonstrated that HT reduced iNOS expression (25 to 100 µM) [128], known for its inflammatory, and oxidative properties in monocytes.

### 10.5. Hydroxytyrosol and Cardiac Dysfunction

All of the cardiovascular risk factors of MetS are associated with increased risk of heart failure. HT was revealed as cardiac protective after olive oil consumption. Bayram et al. [136] have found that female SAMP8 mice fed with a Western diet enriched with a high-polyphenol content (mainly tyrosol (20.8 mg/kg oil) and hydroxytyrosol (18.9 mg/kg oil)) had lower TBARS levels in cardiac muscle. Alterations of cardiac function were correlated with cardiac remodeling leading to blood pressure increase thus raising CVD. Mnafgui et al. [137] showed that a HT supplementation (2 and 5 mg/kg/day × 1 week) in a rodent CVD model leads to a reduction of heart weight and heart weight/body weight ratio. These morphological changes were followed by the reductions of SBP, DBP and mean arterial blood pressure, heart rate and ST segment elevation. Moreover, these authors [137] found an increase of lactate dehydrogenase and creatine kinase protein expressions showing an enhancement of glucose consumption, probably producing higher ATP. Granados-Principal et al. [106] found in cardiac tissue that HT (0.5 mg/kg, 5 days/week × 6 weeks) increases expression of mitochondria complexes 1, 2 and 3 and reduces specific markers of oxidative damages to proteins. These HT beneficial effects on cardiac function are probably due to its antioxidative properties (0.1 and 10 µg/mL) [109], since HT was not found in rat heart (1, 10 and 100 mg/kg) [138].

## 11. Functional Applications in Food Processing

Nowadays, several antioxidants are used to reduce food oxidation and thus extend shelf life. Adding 100 mg/kg HT in frankfurters was effective against lipid oxidation during storage, to a greater extent than a mix of butylated hydroxyanisole/butylated hydroxytoluene [139]. Nieto et al. [140] also showed a reduced lipid oxidation in sausages with HT (50 ppm). Thus HT added in fat used could help to maintain the nutritional and sensorial qualities of processed food products.

## 12. Limitations of Experimental Studies

Ex vivo and in vitro studies with HT are well documented, but question the extrapolation to human relevancy. In fact, high concentrations were usually used in cell models [22,111,135], certainly due to the oxidation of HT [141]. HT has been tested as a sole molecule, and not with other antioxidant compounds (phenolic compounds, tocopherols, carotenoids and vitamin C) that occur in human context. Moreover, the influence of food matrix on the bioactivity of HT is avoided in most of experimental studies.

## 13. Conclusions

**Figure 1:**
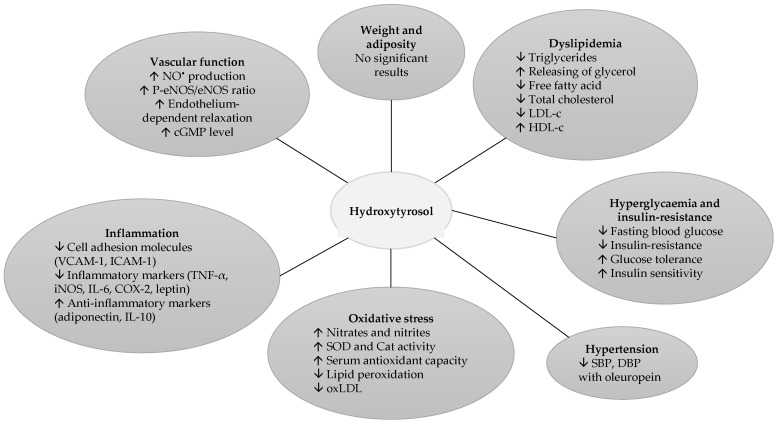
Effect of hydroxytyrosol on metabolic syndrome-associated complications and metabolic syndrome. ↑: Increase in; ↓: Decrease in. LDL-c: Low-Density Lipoprotein-cholesterol; HDL-c: High Density Lipoprotein-cholesterol; SBP: Systolic Blood Pressure; DBP: Diastolic Blood Pressure; SOD: Super Oxide Dismutase; Cat: Catalase; oxLDL: oxidized Low-Density Lipoprotein; VCAM-1: Vascular Cell Adhesion Molecule-1; ICAM-1: Intercellular Adhesion Molecule-1; TNF-α: Tumor Necrosis Factor alpha; iNOS: inducible Nitric Oxide Synthase; IL-6: Interleukin-6; COX-2: Cyclooxygenase-2; IL-10: Interleukin-10; NO^•^: Nitric Oxide; P-eNOS/eNOS: Phosphorylated Endothelial Nitric Oxide Synthase/endothelial Nitric Oxide Synthase ratio; cGMP: cyclic Guanosine Monophosphate.

To conclude, the beneficial effects of HT were extensively studied in rodent experiments and clinical trials evidencing the role of HT in the reduction of MetS and its associated complications, which are briefly presented in Figure 1. Both experimental and clinical studies demonstrated that HT reduced oxidative stress and inflammation, thus altering positively MetS key components. However, contradictory results for obesity were reported, probably resulting from differences in the study design, administered doses and type of animals. Moreover, actually, no experiment assesses the impact of pure HT on blood pressure in normotensive or hypertensive subjects. In this sense, larger and more experimental studies and clinical trials are needed.

The role of dietary polyphenols in human health depends largely on their bioavailability and, because HT bioavailability is reduced, with systemic concentration ranging in the nanomolar levels, it could be expected that HT exerts its effects directly in the gastrointestinal tract before being absorbed. Indeed, the concentration of phenolic compounds may reach the millimolar range. In this sense, modulation of gut microbiota could be the most remarkable local effect exerted by HT. Recently, another component of the Mediterranean diet, resveratrol, has been shown to modulate positively gut microbiota enhancing glucose tolerance in a mice model of obesity [142] and also through a duodenal Sirt-1 pathway into the hypothalamus [143] enhancing hypothalamic insulin-resistance. Sirt-1 is an energy sensitizer upregulating antioxidative and anti-inflammatory gene expression and improving mitochondrial biogenesis through PGC-1α. Direct effects of HT on duodenal Sirt-1 pathway cannot be excluded.

## Abbreviations

The following abbreviations are used in this manuscript:

AMPK	Adenosine monophosphate kinase
ATP	Adenosine triphosphate
Cat	Catalase
CVD	Cardiovascular diseases
eNOS	Endothelial nitric oxide synthase
EVOO	Extra-virgin olive oil
HDL	High-density lipoprotein
HFD	High Fat Diet
IL	interleukin
iNOS	Inducible nitric oxide synthase
LDL	Low density lipoprotein
MUFA	Monounsaturated fatty acid
NO	Nitric oxide
Nrf2	Nuclear factor 2
OLE	Oleuropein
P-eNOS	Phosphorylated endothelial nitric oxide synthase
PGC1α	Peroxisome proliferator-activated receptor gamma coactivator 1-alpha
PUFA	Polyunsaturated fatty acid
ROS	Reactive oxygen species
SFA	Saturated fatty acid
SOD	Superoxide dismustase
TBARS	Thiobarbituric acid reactive substances
TNF-α	Tumor necrosis factor-alpha
VCAM-1	Vascular adhesion molecule-1
VOO	Virgin olive oil
